# Numerical simulations on shear behaviour of rock joint network under constant normal stiffness conditions

**DOI:** 10.1371/journal.pone.0284598

**Published:** 2023-04-21

**Authors:** Guansheng Han, Jiahao Xiang, Zhijing Chen, Zhangjianing Cheng, Yu Zhou, Qiongqiong Tang, Yuan Gao

**Affiliations:** 1 Key Laboratory of Rock Mechanics and Geohazards of Zhejiang Province, Shaoxing University, Shaoxing, Zhejiang, China; 2 College of Civil Engineering, Tongji University, Shanghai, China; 3 School of Transportation and Civil Engineering, Nantong University, Nantong, China; 4 Nantong Key Laboratory of Intelligent Civil Engineering and Digital Construction, Nantong University, Nantong, China; University of Science and Technology Beijing, CHINA

## Abstract

In this study, the numerical direct shear tests were conducted to investigate the shear mechanical properties of joint networks under constant normal stiffness (CNS) boundary conditions. The influence of random joint number on shear stress (*τ*), dilation (normal displacement, *δ*_v_) and normal stress (*σ*_*n*_) of rock mass were studied quantitatively with fixed main slip surface. At the same time, the internal stress evolution process and failure process were analyzed. The results reveal that the number of random joints (*γ*) has little effect on the shear and normal stresses. The normal displacement of the sample generally decreases as the number of random joints increases. In addition, the normal displacement of the specimen is absorbed by the random joints when the number of random joints in the specimen increases to a certain level: when *γ* is greater than 6 and the shear displacement (*μ*) reaching 10 mm, the specimen exhibits shear contraction. Therefore, the internal random joints mainly control the failure mode and dilatancy performance of the specimen, while the main joint of the rock controls the shear stress of the specimen.

## Introduction

Shear failure is a typical failure form of the surrounding rock in high-stress underground engineering [1]. The shear cracks developed in the surrounding rock of high-stress underground engineering cut the complete surrounding rock into blocks [2]. Under the action of the redistributed stress of the surrounding rock, these blocks undergo shear slippage along the fracture surface, resulting in disasters, such as large deformation of the roadway and damage to the supporting structure [3]. The shear performance of a rock mass is strongly affected by the presence of joints [4]. Recognizing the significance of its evaluation for underground engineering, many researchers have focused on the shear characteristics of the joint surfaces since the 1960s. For deep underground rock engineering, when a shear-slip failure occurs, the normal stress acting on the rock mass would continue to increase due to the restriction of the surrounding rock in the underground or rock bolts and cannot freely shear dilatation [5–7]. Therefore, constant normal load (CNL) boundary conditions can no longer meet the site requirements [8], and constant normal stiffness (CNS) boundary conditions that are more in line with the actual situation should be adopted [9]. Therefore, it is of great significance to investigate the shear behaviour of joints under CNS boundary conditions by designing shear tests that conform to the stress state of surrounding rock in deep underground rock engineering [10].

To better investigate the direct shear characteristics of jointed rock mass, some scholars have carried out experimental researches. Indraratna et al. [11, 12] carried out research on the shear behavior of infilled joints under the boundary conditions of CNS based on the world’s first independently developed CNS shear equipment. The research results showed that even if a thin layer of filler was added, the shear strength of the joint would be significantly reduced. Since the equipment of Indraratna et al. uses spring to simulate the normal stiffness, their equipment cannot realize the free switching of the normal stiffness. Subsequently, Jiang et al. [13, 14] developed the first servo-controlled CNC shearing machine, which realized the free switching of boundary conditions. Jiang et al. found that the normal stiffness has a significant influence on the mechanical behavior of joints during the shear process based on the shear tests conducted using the servo-controlled CNC shearing machine. Xia et al. [15] developed a shear-flow coupling experiment system for rock joints under CNS boundary conditions, and studied the hydraulic coupling characteristics in the shear process. In addition, the shear behavior of joints under CNS boundary conditions has been further studied and developed with the support of the above-mentioned equipment [16–19].

In summary, previous studies have reflected the shear mechanical properties of rock joints under CNS boundary conditions, including shear strength, dilatancy characteristics and joint surface damage. However, most of the studies on rock joint shear under CNS boundary conditions [20] are limited to the study of single joints due to the test conditions and difficulties in specimen preparation. But joints do not exist in isolation in rock masses, and they are often widely distributed within the rock masses in the form of networks [21]. Since indoor experiments have great difficulties in preparing joint network rock masses, numerical simulation studies are needed to fill the gaps in this research field. The numerical simulation method (the Finite Element Method, FEM or the Distinct Element Method, DEM) [22], laboratory test and theoretical analysis are known as the three main analysis methods. Particularly, the repeatability, simplicity, convenience and diversity of numerical simulation methods play an important role in the field of studying deep underground engineering [23]. For example, Xu [24] and Saadat M [25] used the particle flow code (PFC) to study sawtooth filling joints with different normal stresses, different undulation angles and different filler thicknesses. And the shear mechanical properties and crack propagation phenomena of the sawtooth filling joints were reproduced. Asadi M S [26, 27], Bahaaddini [28, 29] and Gutiérrez-Ch J.G [30] investigated the mechanical properties of shear damage and the evolution of micro-cracks during the shear process of serrated joints with different geometrical characteristics at different initial normal stresses (*σ*_*n*0_), different shear directions by using PFC. However, there are few numerical simulation studies on the shear properties of joint network under CNS conditions.

Therefore, we adopted an innovative cyclic assignment method of the smooth joint model (SJM) [31] in this study to overcome the failure problem of the joint model during the shear test. The numerical simulation model of the jointed rock mass under CNS boundary conditions was firstly established to investigate the shear behavior of the joint network under the CNS boundary condition. Then, the influence of random joint number on shear stress, normal stress and normal displacement of jointed rock mass were analyzed.

## Numerical simulation of direct shear test in PFC

### Establishment of rock single joint model

In previous studies, the numerical simulation research of joint shear mainly adopted the following two methods: 1) Bond deletion method. This method represents the joint surface by removing the particle bonding within a certain distance on both sides of the joint trajectory. Although this method is simple, the resulting joint surface will cause joint roughness at the microscopic scale, resulting in interlocking particles on the joint surface will make the simulation results far from the actual results [32]. 2) The smooth joint model method. It is widely used in PFC to simulate the mechanical behavior of rock weak surface and the direct shear test. However, the direct application of the SJM cannot completely and correctly simulate the shear behavior of real joints. For example, Bahaaddini et al. [28, 29] found that even a plane joint with a SJM can only correctly simulate the real shear strength when the shear displacement is smaller than the minimum particle radius. When the shear displacement is larger than the minimum particle radius, the simulated strength is greatly biased high or low. This is because the particles on both sides of the joint surface cannot be correctly identified during the shear process when the SJM is directly applied, and the resulting stress concentration in local particle contact will cause distortion of the shear stress. Based on the above deficiencies, Bahaaddini et al. adopted a new shear box generation method and an SJM assignment method, and achieved good application results [33–37]. In this work, the numerical simulation of joint shear under CNS boundary conditions is also studied based on this method.

**Fig 1:**
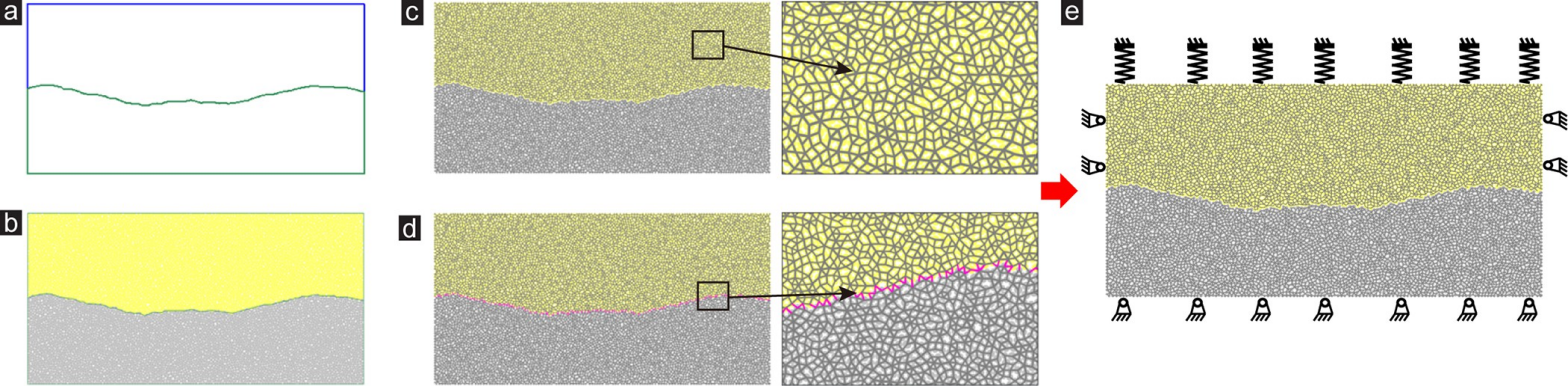
Setup of particle flow model of rock joints under CNS conditions: (a) Initial shear box assembly, (b) Generation of particles, (c) Apply the Parallel Bond Model, (d) Assignment Smooth Joint Model, (e) Application of CNS conditions.

Taking the example of a single joint (the contour curves of standard joint roughness coefficient (JRC) were proposed by Barton and Choubey [38], JRC = 10–12), the establishment steps of a single joint model in deep rock are as follows:

Initial shear box assembly (see Fig 1A): Use the wall command in PFC to generate the upper and lower parts of the frictionless wall separately. At the same time, the geometry command is used to import complex joint contours. Since only one side of a wall is activated and effective, the generation of a complex joint profile requires the creation of two walls.

Generation of particles (see Fig 1B): Uniformly distributed particle aggregates satisfying a certain pore density are formed in the upper and lower walls respectively. The minimum radius (*R*_min_) is 0.5 mm and the maximum radius (*R*_max_) is 0.75 mm. Such particle size not only satisfies the calculation requirements of computer capacity but also has relatively accurate and stable calculation results. This step requires sufficient time to make the generated particle assembly reach a static equilibrium state.

Apply the Parallel Bond Model (see Fig 1C): In this step, the floating particles (particles with less than three contacts) in the particle assembly generated in step ‘Generation of particles’ are deleted first to improve computational efficiency. Then the parallel bond model (PBM) is used to simulate the mechanical behavior of the intact rock part. And the linear contact model (LM) was assigned between particles and walls.

Assignment Smooth Joint Model (see Fig 1D): The application of the SJM is the core of this modeling approach. Firstly, the joint surface between the up and down particle groups is identified. Then the smooth joint model is continuously assigned to the joint surface during the application of normal stress and shear, and the smooth joint direction at each contact is consistent with the overall joint. This approach can ensure that there must be a smooth joint model where the joint surfaces are in contact during the shear process. Otherwise, the effectiveness of the joint model would cause distortion of the simulated shear strength as the *μ* increases.

The numerical model and boundary conditions are shown in Fig 1A–1E. Corresponding calibrated micro-parameters are listed in Table 1.

**Table 1:** Micromechanical parameters of PFC numerical model.

	Micro-parameters	Values
Particle and linear contact	Radius of minimum particle, *R*_min_ (mm)	0.5
	Particle-size ratio, *R*_min_ / *R*_max_	1.5
	Particle density, *ρ* (g/cm^3^)	1546
	Coefficient of particle friction, *υ*	0.3
	Particle linear effective modulus, *E*_c_ (GPa)	3.98
	Linear contact normal to shear stiffness ratio, *K*_n_ / *K*_s_	1.25
Parallel bond model	Parallel bond effective modulus, *E*_c_* (GPa)	3.98
	Parallel bond normal to shear stiffness ratio, *K*_n_* / *K*_s_*	1.25
	Parallel bond cohesion, c* (MPa)	12.0
	Parallel bond tensile strength, *σ*_t_* (MPa)	5.10
	Parallel bond friction angle, φ* (°)	0.0
Smooth joint model	Smooth-joint contact normal stiffness, sj_kn (GPa/m)	100
	Smooth-joint contact shear stiffness, sj_ks (GPa/m)	50
	Smooth-joint contact friction coefficient, *υ*_*sj*_	0.45

### Calibration of parameters

In the physical test, the super rock brand plaster produced in Japan is used as the rock-like materials. The basic mechanical properties of the plaster are as follows: the optimal water mixing ratio is 1:0.2; The hardening time is 10 minutes; the expansion rate is 0.09% in 2 hours; the expansion rate is 0.13% in 24 hours; the uniaxial compressive strength after 1 hour is 50 MPa. Therefore, the samples cast with plaster can better simulate the real rock situation.

**Fig 2:**
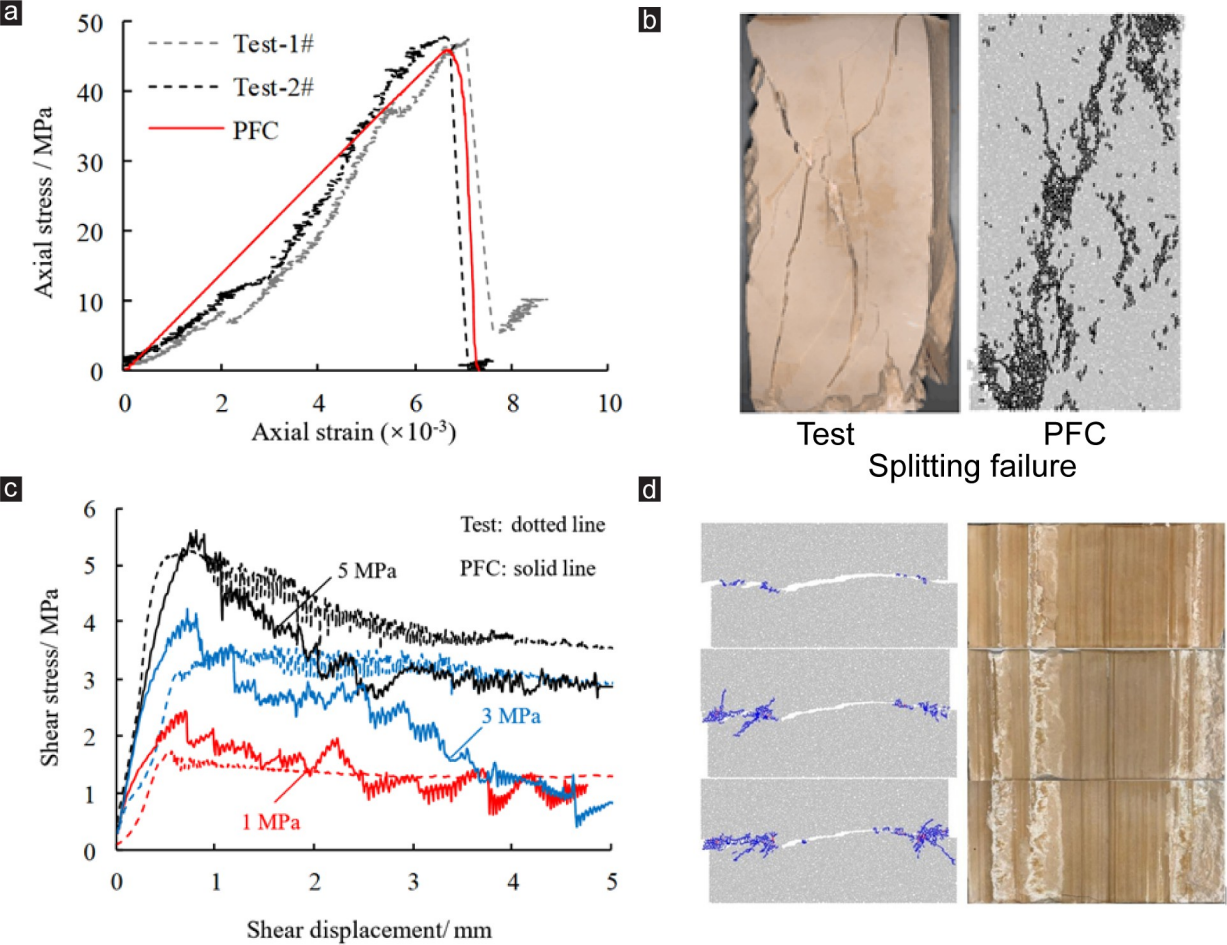
Check of parameters: (a) and (b) are uniaxial compression tests, (c) and (d) are CNS shear tests.

First, PBM and LM were calibrated and validated by uniaxial compression tests (see Fig 2A and 2B), the results show that the uniaxial compressive strength, peak strain, Young’s modulus and Poisson’s ratio of the complete specimen in the numerical simulation test are basically consistent with the physical model test. Then SJM was calibrated by CNS shear test (see Fig 2C and 2D). When JRC = 10 ~ 12 and *σ*_*n*0_ is 1, 3 and 5 MPa respectively, the shear stress-shear displacement curve and the final failure mode of the specimen in the numerical simulation test are basically consistent with the physical test. Therefore, we believe that the micro-parameters adopted in the numerical simulation experiments are reasonable.

### Establishment and numerical calculation scheme of rock random joint network model

**Fig 3:**
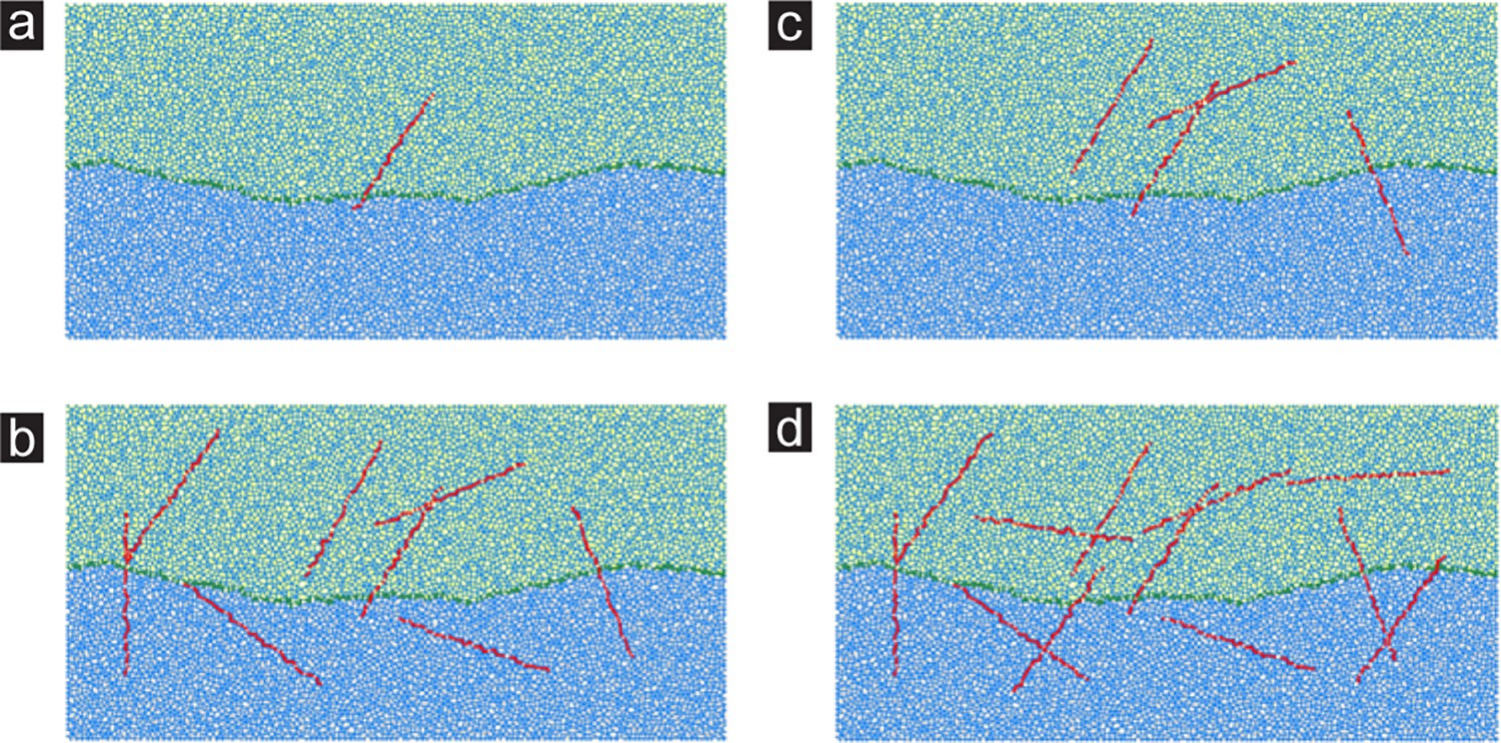
Diagram of stochastic joint network model of rock: (a) *γ* = 1, (b) *γ* = 4, (c) *γ* = 8, (d) *γ* = 12.

The size of the rock random joint network model was designed as 200mm×100mm. In order to simplify the calculation model, the rock random joint network model mentioned in this paper consists of a main joint and random joints. Standard JRC Curve with JRC = 10 ~ 12 for the main joint. The random joints are through joints, which are simulated by deleting particles at specific positions. The length is set to 50 mm, and their spatial positions are randomly generated and conform to the Gaussian function distribution. In addition, the number of random joints is the research variable of this paper. The number of random joints is expressed by *γ*, and a total of 12 working conditions (*γ* increases from 1 to 12) are studied. A partial rock random joint network models are shown in Fig 3.

The specific test scheme is as follows: the initial normal stress is set to 1MPa, the normal stiffness (*k*_*n*_) is set to 3 GPa/m, the maximum shear displacement (10 mm) is 5% of the specimen length (200 mm) and the shear rate is set to 0.1 m/s. (The loading speed of 0.1’m/s’ can fully simulate the quasi-static loading conditions; when the loading speed is 0.1’m/s’, it takes about 3750 steps to produce 1mm shear displacement).

## Results and discussion

### Influence of random joint number on shear behaviours

**Fig 4:**
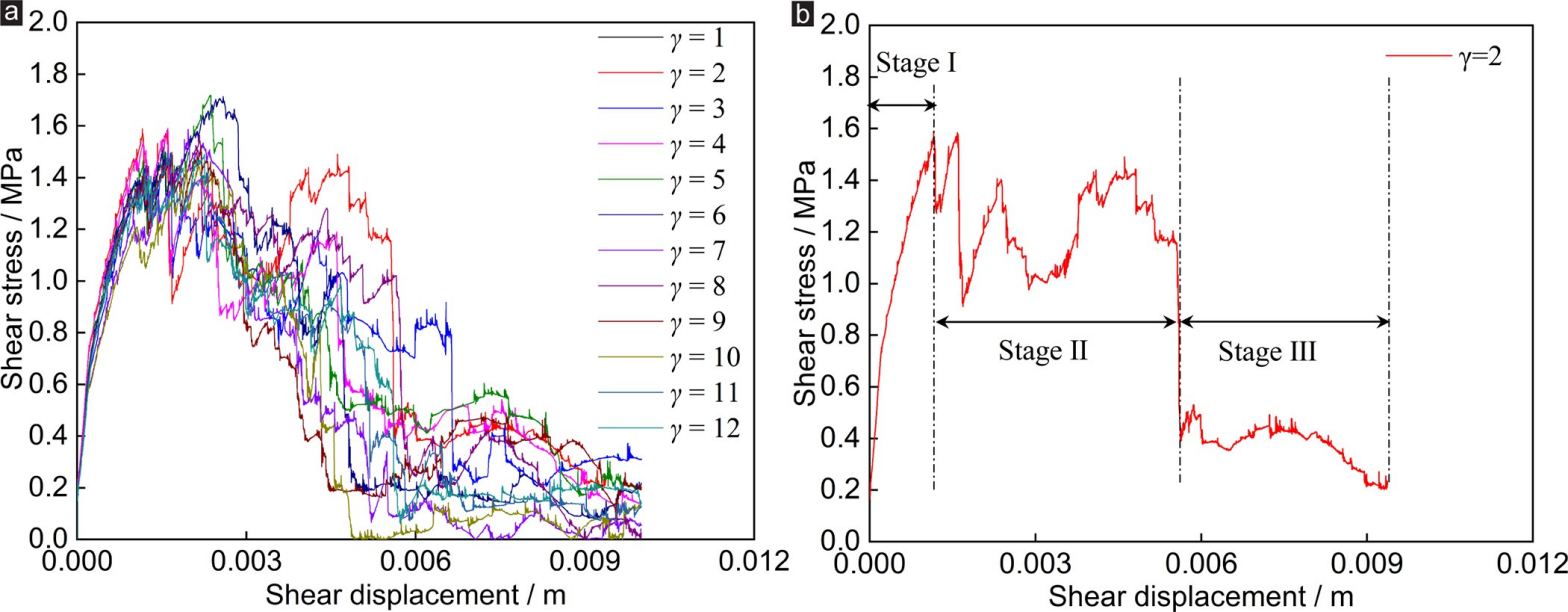
Effect of *γ* on shear stress of rock joints under CNS conditions: (a) shear stress vs. shear displacement, (b) shear stress vs. shear displacement of *γ* = 2.

In terms of shear stress: under CNS boundary conditions, the variations of shear stress of rock random joint network specimens under different random joint numbers are shown in Fig 4A. It can be seen from Fig 4A that the number of random joints has little effect on the shear stress of the specimen. The cures of shear stress versus (vs.) shear displacement are mostly coincides while the number of random joints increases from 1 to 12. It can be seen that the main joint controls the shear stress of the sample to a large extent.

The curve of shear stress vs. shear displacement can be roughly divided into rapid rise stage, serrated decline stage and residual stage. Taking *γ* = 2 as an example (Fig 4B), when the *μ* increases from 0 to 1.16 mm, the *τ* of the sample reaches the first peak value of 1.59 MPa (Stage I). When the *μ* is between 1.16 mm and 5.6 mm, the shear stress of the specimen appears multiple peaks successively (all of which are lower than the first peak in value), and the peak intensity gradually decreases (Stage II). When the *μ* increases from 5.6 mm to 10 mm, the shear stress of the specimen fluctuates within a range of fewer than 0.53 MPa (Stage III).

On the one hand, the shear stress of the specimen decreases due to the shearing of the convex joint surface during the shear test. On the other hand, the shear stress of the specimen increases due to the constraint of normal stiffness. The two factors compete with each other, so the phenomenon of the serrated decrease in shear stress appears.

**Fig 5:**
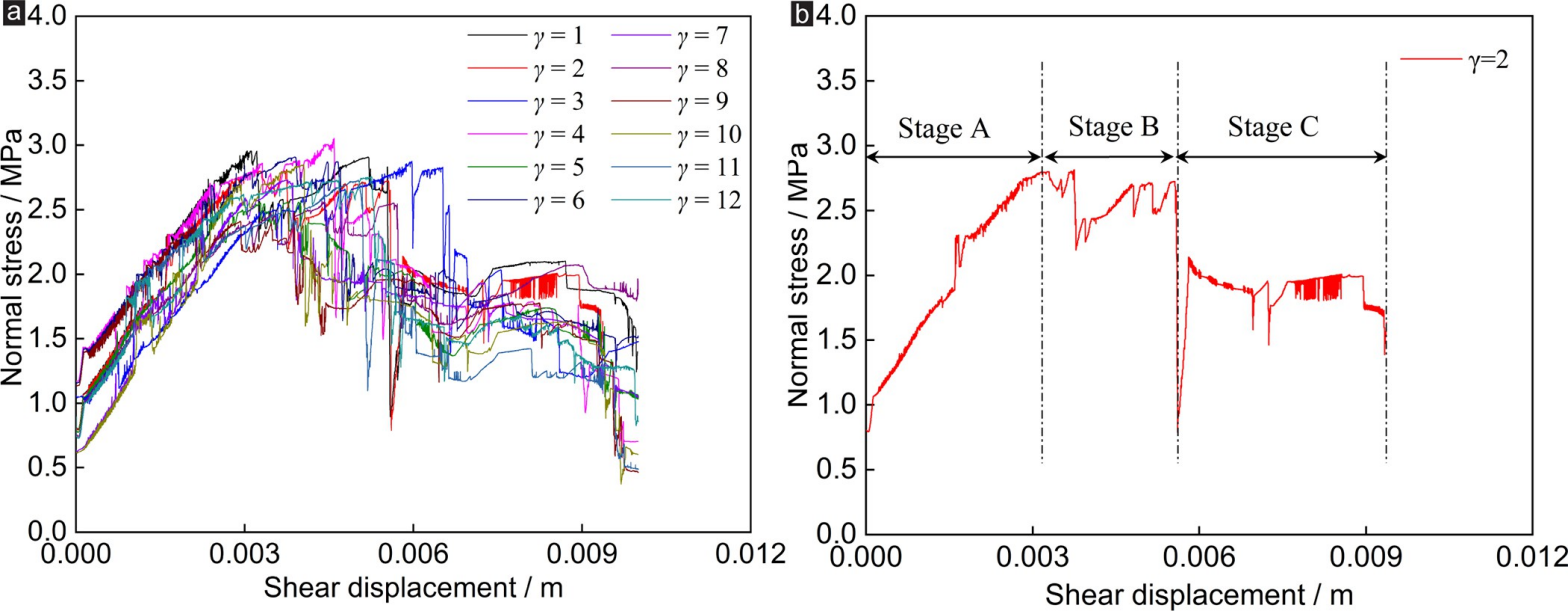
Effect of *γ* on normal stress of rock joints under CNS conditions: (a) normal stress vs. shear displacement, (b) normal stress vs. shear displacement of *γ* = 2.

In terms of normal stress: the evolutions of *σ*_*n*_ of rock joint network specimens under different number of random joints under CNS boundary conditions are shown in Fig 5A. It can be seen from Fig 5A that the number of random joints has little effect on the normal stress of the specimen. The trends of the curve of normal stress vs. shear displacement are basically consistent when the number of random joints increases from 1 to 12.

With the increase of *μ*, the normal stress of the specimen can be divided into rapid rise stage, stable stage and residual stage. Also taking *γ* = 2 as an example (Fig 5B): Stage A: When the *μ* increases from 0 to 3.3 mm, the normal stress of the specimen increases rapidly from 1 Ma (*σ*_*n*0_) to the first peak value of 2.7 MPa. Stage B: When the *μ* increases from 3.3 to 5.6 mm, the normal stress of the specimen fluctuates in the range of 2.2 MPa to 2.7 MPa. Stage C: When the *μ* increases from 5.6 to 10 mm, the normal stress of the specimen fluctuates in the range of 1.5 MPa to 2.1 MPa. In the CNS shear test, the normal stress of the specimen is generally higher than the initial normal stresses due to the shear dilatancy of joints (Eq 1) [13].


$$\sigma_{n}=\sigma_{n0}+k_{n}\times \delta_{v}$$ (1)

Where *σ*_*n*_ is the real-time normal stress of the specimen, *σ*_*n*0_ is the initial normal stress set by the test, *k*_*n*_ is the normal stiffness set by the test, and *δ*_v_ is the normal displacement increment.

**Fig 6:**
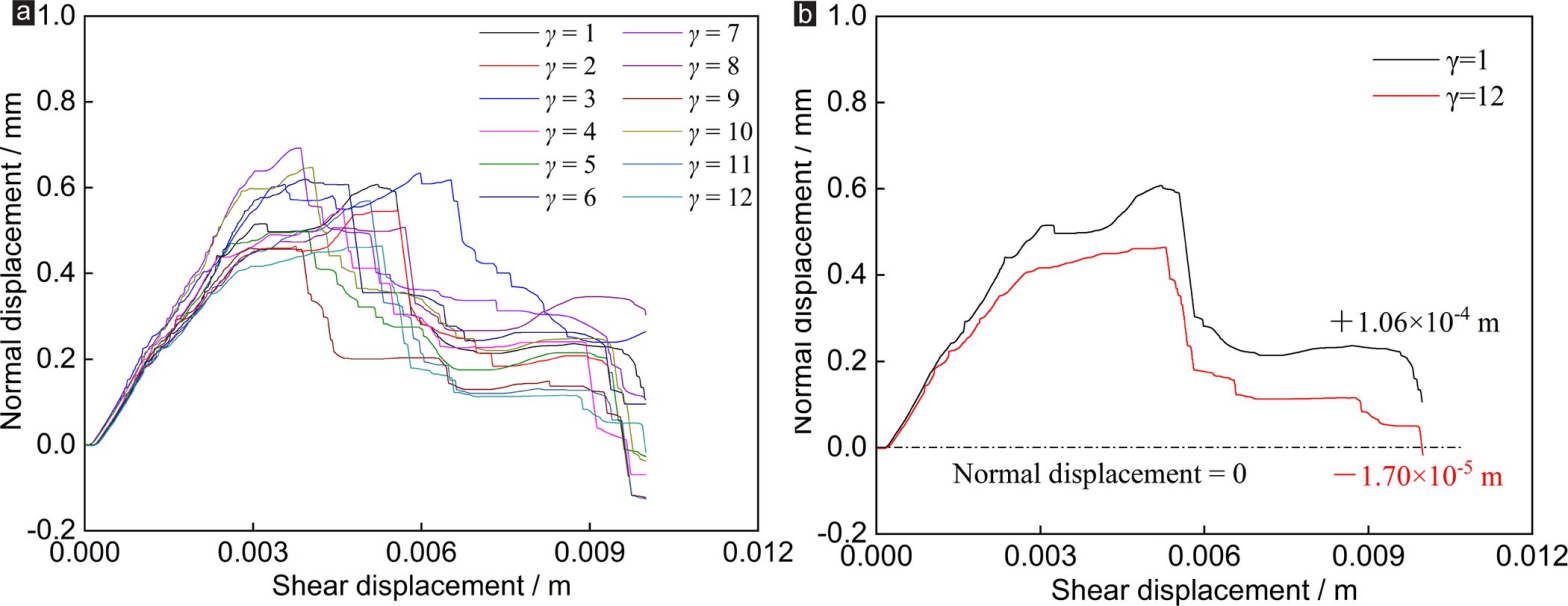
Effect of *γ* on normal displacement of rock joints under CNS conditions: (a) normal displacement vs. shear displacement, (b) normal displacement vs. shear displacement of *γ* = 1 and *γ* = 12.

In terms of normal displacement: the variations of normal displacement of rock joint network specimens under different numbers of random joints under CNS boundary conditions are shown in Fig 6A. With the increase of *μ*, the normal displacement of the specimen can be divided into rapid increase and gradual decrease stages. As the number of random joints increases, the normal displacement of the specimen decreases gradually. It is noteworthy that the specimen exhibits shear contraction when the *μ* is equal to 10 mm when the number of random joints in the specimen is greater than 6. Taking *γ* = 1 and *γ* = 12 as examples (Fig 6B): during the whole shear test, the normal displacement of the specimen with *γ* = 1 is higher than the specimen with *γ* = 12; When the *μ* is equal to 10 mm, the final state of the sample with *γ* = 1 shows shear dilatancy, and the dilatancy amplitude is +1.06×10–4 m. The final state of the sample with *γ* = 12 shows shear contraction, and the shear contraction amplitude is -1.70×10–5 m. The above phenomenon shows that after the number of random joints in the specimen reaches a certain extent, the dilatancy generated during the shear process of the specimen will be absorbed by the random joints.

### Micromechanical behavior: Evolution of inner stress and the failure modes

**Fig 7:**
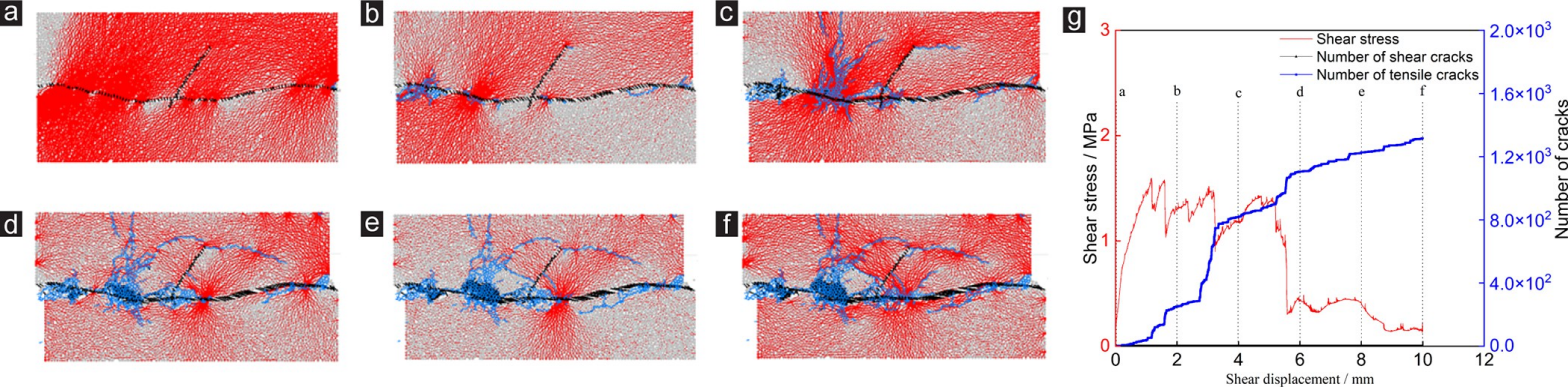
Evolution process of internal stress in specimens for *γ* = 1. (a) *μ* = 0.2 mm, (b) *μ* = 2 mm, (c) *μ* = 4 mm, (d) *μ* = 6 mm, (e) *μ* = 8 mm, (f) *μ* = 10 mm, (g) shear stress-shear displacement and number of cracks-shear displacement.

**Fig 8:**
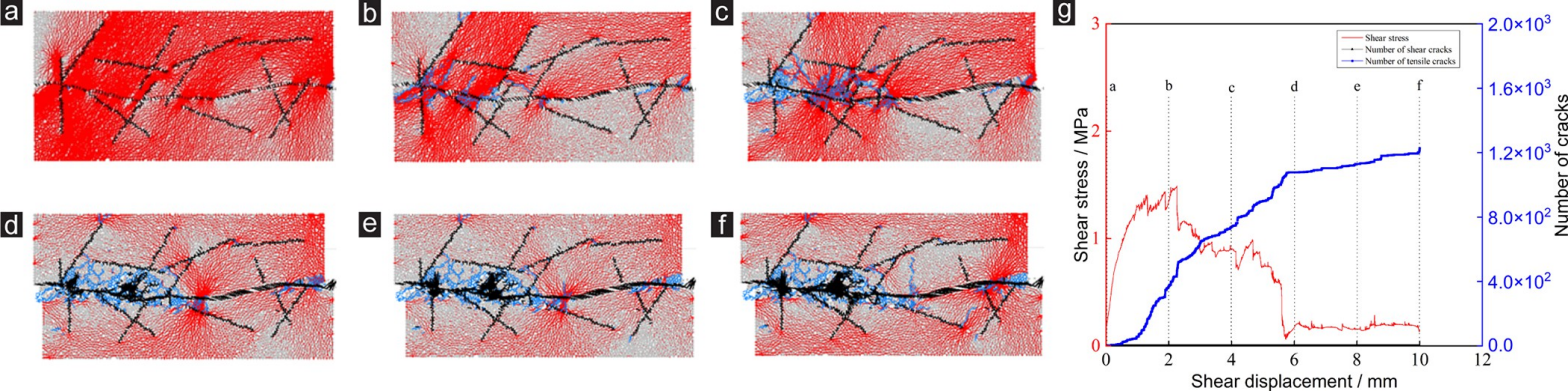
**Evolution process of internal stress in specimens for *γ* = 12.** (a) *μ* = 0.2 mm, (b) *μ* = 2 mm, (c) *μ* = 4 mm, (d) *μ* = 6 mm, (e) *μ* = 8 mm, (f) *μ* = 10 mm, (g) shear stress-shear displacement and number of cracks-shear displacement.

The inner stress evolution and failure mode of the specimen during the shear process are shown in Figs 7 and 8. Figs 7 and 8 are the inner stress evolution process and failure process of the rock joint network specimens corresponding to *γ* = 1 and *γ* = 12. Among them, (a) ~ (f) are the stress distribution of the specimen at the *μ* is 0.2, 2, 4, 6, 8 and 10 mm, respectively. The red area indicates the compression area, and the blue area indicates the crack. And (g) is the relationship between shear stress, tensile crack and shear displacement.

The inner stress distribution of the specimen corresponding to *γ* = 1 is shown in Fig 7. When the *μ* is small (*μ* = 0.2 mm), the interior of the specimen is compressed. There is no obvious stress concentration area and obvious microcrack. When the *μ* increases to 2 mm, microcracks appear at the protruding position of the joint, and the stress concentration area appears nearby. With the increase of *μ*, the internal compression area of the specimen decreases gradually and the number of microcracks increases significantly. The specimen is gradually destroyed. When the *μ* reaches the maximum value (10 mm), the failure area of the specimen is concentrated near the main joint. When *γ* = 1, the existence of random joints has little effect on the failure mode of the specimen. In addition, the microcracks inside the specimen are mainly tensile cracks.

The inner stress distribution of the specimen corresponding to *γ* = 12 is shown in Fig 8. When the *μ* is equal to 2 mm, microcracks are generated inside the specimen at the protrusion position of the main joint and the tip of the random joint. With the increase of *μ*, the number of microcrack areas is increasing, and the number of microcracks is also increasing. It is worth noting that the locations of cracks are near the main joint or the tip of the random joint. When the *μ* is equal to 3 mm, the *τ* of the specimen begins to decrease obviously, and the number of tensile cracks inside the specimen increases rapidly.

Meanwhile, the shear resistance of the specimen is affected by the rapid development and penetration of the microcracks. When the *μ* is equal to 6 mm, the specimen almost loses the shear resistance. When the *μ* continues to increase, the number of tensile cracks in the specimen increases slowly. Some tensile cracks will be generated at the tips of random joints, and these tensile cracks will continue to develop and penetrate during the shear test. The existence of random joints affects the failure mode of the specimen to a certain extent.

## Conclusions

The shear effect of rock random joint network specimens under CNS boundary conditions was studied by numerical simulation. The reproduce of CNS boundary condition, the rough joints and the cyclic assignment of joints have been well realized through the self-developed PFC2D code. The mainly obtained conclusions are as follows:

1. The number of random joints has little effect on the shear stress and normal stress of the sample. The variation curve of shear stress with shear displacement can be roughly divided into rapid rise stage, serrated decline stage and residual stage. With the increase of *μ*, the normal stress of the specimen can be divided into rapid rise stage, stable stage and residual stage. As the number of random joints increases, the normal displacement of the specimen generally decreases.
2. The shear dilatancy generated during the shear process of the specimen will be absorbed by the random joints after the number of random joints in the specimen reaches a certain extent. For example, when the *δ*_h_ is 10 mm, the final state of the sample with *γ* = 1 shows shear dilatancy, and the shear dilatancy amplitude is +1.06×10–4 m. And the final state of the sample with *γ* = 12 shows shear contraction, and the shear contraction amplitude is -1.70×10–5 m.
3. In the shear test of rock random joint network, the random joints inside the rock mainly affect the failure mode and the shear dilatancy performance of the specimen, while the main joint of the rock controls the shear stress of the specimen.

## References

[1] Zhao J, Feng X, Zhang X, Zhang Y, Zhou Y, Yang C. Brittle-ductile transition and failure mechanism of Jinping marble under true triaxial compression. Engineering Geology. 2018;232:160–70. doi: 10.1016/j.enggeo.2017.11.008

[2] Yang S, Chen M, Jing H, Chen K, Meng B. A case study on large deformation failure mechanism of deep soft rock roadway in Xin’An coal mine, China. Engineering Geology. 2017;217:89–101. doi: 10.1016/j.enggeo.2016.12.012

[3] Jiao Y, Song L, Wang X, Adoko AC. Improvement of the U-shaped steel sets for supporting the roadways in loose thick coal seam. International Journal of Rock Mechanics and Mining Sciences. 2013;60:19–25. doi: 10.1016/j.ijrmms.2012.12.038

[4] Liu H, Yuan X. A damage constitutive model for rock mass with persistent joints considering joint shear strength. Canadian Geotechnical Journal. 2015;52(8):1136–43. doi: 10.1139/cgj-2014-0252

[5] Jiang Y, Li B, Tanabashi Y. Estimating the relation between surface roughness and mechanical properties of rock joints. International Journal of Rock Mechanics and Mining Sciences. 2006;43(6):837–46. doi: 10.1016/j.ijrmms.2005.11.013

[6] Han G, Zhou Y, Liu R, Tang Q, Wang X, Song L. Influence of surface roughness on shear behaviors of rock joints under constant normal load and stiffness boundary conditions. Natural Hazards. 2022;112(1):367–85. doi: 10.1007/s11069-021-05185-8

[7] Thirukumaran S, Indraratna B, Brown E, Kaiser PK. Stability of a rock block in a tunnel roof under constant normal stiffness conditions. Rock Mechanics and Rock Engineering. 2016;49:1587–93. doi: 10.1007/s00603-015-0770-6

[8] Zofka A, Maliszewski M, Bernier A, Josen R, Vaitkus A, Kleizienė R. Advanced shear tester for evaluation of asphalt concrete under constant normal stiffness conditions. Road Materials and Pavement Design. 2015;16(sup1):187–210. doi: 10.1080/14680629.2015.1029690

[9] Indraratna B, Thirukumaran S, Brown E, Zhu S-P. Modelling the shear behaviour of rock joints with asperity damage under constant normal stiffness. Rock Mechanics and Rock Engineering. 2015;48:179–95. doi: 10.1007/s00603-014-0556-2

[10] Han G, Xiong F, Zhou Y, Song L, Wang X. Research Progress on Shear Characteristics of Rock Joints under Constant Normal Stiffness Boundary Conditions. Shock and Vibration. 2021;2021:1–6. doi: 10.1111/ffe.13973

[11] Indraratna B, Haque A, Aziz N. Shear behaviour of idealized infilled joints under constant normal stiffness. Geotechnique. 1999;49(3):331–55. doi: 10.1680/geot.1999.49.3.331

[12] Indraratna B, Haque A. Shear behaviour of rock joints. 2000.

[13] Jiang Y, Xiao J, Tanabashi Y, Mizokami T. Development of an automated servo-controlled direct shear apparatus applying a constant normal stiffness condition. International Journal of Rock Mechanics and Mining Sciences. 2004;41(2):275–86. doi: 10.1016/j.ijrmms.2003.08.004

[14] Jiang Y, Wang G, Li B, Zhao X. Experimental study and analysis of shear-flow coupling behaviors of rock joints. Chinese Journal of Rock Mechanics and Engineering. 2007;26(11):2253–9.

[15] XIA C, QIAN X, GUI Y, Zhuang X, Yu Q, Zhang L. A novel multi-functional shear-flow coupled test system for rock joints and its application. Chinese Journal of Rock Mechanics and Engineering. 2018;37(10):16–28. doi: 10.13722/j.cnki.jrme.2018.0359

[16] Ghazvinian A, Taghichian A, Hashemi M, Mar’Ashi S. The shear behavior of bedding planes of weakness between two different rock types with high strength difference. Rock Mechanics and Rock Engineering. 2010;43:69–87. doi: 10.1007/s00603-009-0030-8

[17] Liu R, Yin Q, Yang H, Jing H, Jiang Y, Yu L. Cyclic shear mechanical properties of 3D rough joint surface under constant normal stiffness (CNS) boundary conditions. Chinese journal of rock mechanics and Engineering. 2021;40(6):1092–109. doi: 10.13722/j.cnki.jrme.2020.1128

[18] Han G, Jing H, Jiang Y, Liu R, Wu J. Effect of cyclic loading on the shear behaviours of both unfilled and infilled rough rock joints under constant normal stiffness conditions. Rock Mechanics and Rock Engineering. 2020;53:31–57. doi: 10.1007/s00603-019-01866-w

[19] Liu J, Wu J, Li X, Zhang H, Song Y. Numerical Simulation on Shear Behavior of Double Rough Parallel Joints Under Constant Normal Stiffness Boundary Condition. Frontiers in Earth Science. 2022;9:1405. doi: 10.3389/feart.2021.819290

[20] Xie S, Lin H, Han Z, Duan H, Chen Y, Li D. A New Shear Constitutive Model Characterized by the Pre-Peak Nonlinear Stage. Minerals. 2022;12(11):1429. doi: 10.3390/min12111429

[21] Oda M. An equivalent continuum model for coupled stress and fluid flow analysis in jointed rock masses. Water resources research. 1986;22(13):1845–56. doi: 10.1029/WR022i013p01845

[22] Jonsén P, Pålsson BI, Tano K, Berggren A. Prediction of mill structure behaviour in a tumbling mill. Minerals Engineering. 2011;24(3–4):236–44. doi: 10.1016/j.mineng.2010.08.012

[23] Hu B, Zhang Q, Li S, Yu H, Wang X, Wang H. Application of numerical simulation methods in solving complex mining engineering problems in dingxi mine, China. Minerals. 2022;12(2):123. doi: 10.3390/min12020123

[24] Xu L, Ren QW, editors. Shear failure mechanism of infilling rock joints and its PFC simulation. Applied Mechanics and Materials; 2015: Trans Tech Publ.

[25] Saadat M, Taheri A. A cohesive discrete element based approach to characterizing the shear behavior of cohesive soil and clay-infilled rock joints. Computers and Geotechnics. 2019;114:103109. doi: 10.1016/j.compgeo.2019.103109

[26] Asadi MS, Rasouli V, Barla G. A bonded particle model simulation of shear strength and asperity degradation for rough rock fractures. Rock Mechanics and Rock Engineering. 2012;45:649–75. doi: 10.1007/s00603-012-0231-4

[27] Asadi M, Rasouli V, Barla G. A laboratory shear cell used for simulation of shear strength and asperity degradation of rough rock fractures. Rock Mechanics and Rock Engineering. 2013;46:683–99. doi: 10.1007/s00603-012-0322-2

[28] Bahaaddini M, Hagan P, Mitra R, Khosravi M. Experimental and numerical study of asperity degradation in the direct shear test. Engineering Geology. 2016;204:41–52. doi: 10.1016/j.enggeo.2016.01.018

[29] Bahaaddini M. Effect of boundary condition on the shear behaviour of rock joints in the direct shear test. Rock Mechanics and Rock Engineering. 2017;50(5):1141–55. doi: 10.1007/s00603-016-1157-z

[30] Gutiérrez-Ch J, Senent S, Melentijevic S, Jimenez R. Distinct element method simulations of rock-concrete interfaces under different boundary conditions. Engineering Geology. 2018;240:123–39. doi: 10.1016/j.enggeo.2018.04.017

[31] Huang D, Wang J, Liu S. A comprehensive study on the smooth joint model in DEM simulation of jointed rock masses. Granular Matter. 2015;17:775–91. doi: 10.1007/s10035-015-0594-9

[32] Pierce M, Cundall P, Potyondy D, Ivars DM, editors. A synthetic rock mass model for jointed rock. 1st Canada-US Rock Mechanics Symposium; 2007: OnePetro.

[33] Lambert C, Buzzi O, Giacomini A. Influence of calcium leaching on the mechanical behavior of a rock–mortar interface: A DEM analysis. Computers and Geotechnics. 2010;37(3):258–66. doi: 10.1016/j.compgeo.2009.09.006

[34] Lambert C, Coll C. A DEM approach to rock joint strength estimate. 2009. hdl.handle.net/10092/8453

[35] Bahaaddini M. Numerical study of the mechanical behaviour of rock joints and non-persistent jointed rock masses. School of Mining Engineering, the University of New South Wales, Sydney, Australia. 2014.

[36] Bahaaddini M, Hagan P, Mitra R, Hebblewhite B. Scale effect on the shear behaviour of rock joints based on a numerical study. Engineering Geology. 2014;181:212–23. doi: 10.1016/j.enggeo.2014.07.018

[37] Bahaaddini M, Hagan P, Mitra R, Hebblewhite B. Parametric study of smooth joint parameters on the shear behaviour of rock joints. Rock Mechanics and Rock Engineering. 2015;48:923–40. doi: 10.1007/s00603-014-0641-6

[38] Barton N, Choubey V. The shear strength of rock joints in theory and practice. Rock mechanics. 1977;10:1–54. doi: 10.1007/BF01261801

